# Life History-Dependent Brain Transcriptomic Signatures in Nothobranchids: Insights into Aging, Neurogenesis, and Life History Evolution

**DOI:** 10.1093/iob/obaf016

**Published:** 2025-04-17

**Authors:** C J Leow, K R Piller

**Affiliations:** Department of Biological Sciences, Southeastern Louisiana University, Hammond, LA 70402, USA; Department of Biological Sciences, Southeastern Louisiana University, Hammond, LA 70402, USA

## Abstract

The African turquoise killifish *Nothobranchius furzeri* is a powerful model organism in aging research. Within the family Nothobranchiidae, a wide range of lifespan is observed in annual, semi-annual, and non-annual life histories. In this study, we examined the brain transcriptomic signatures of adult nothobranchids across life history variations. Our results show that the brain gene expression profiles exhibit strong life history signatures compared to the liver tissue. Semi-annual *Fundulopanchax* species shows upregulation in cell division and mitosis compared to non-annual *Aphyosemion* species. We identified genes related to neurogenesis such as *DNMT3A*, *SOX2*, and *FGF10* that show downregulation in the short-lived annual species compared to other life histories. The Notch signaling pathway is enriched in the non-annual species suggesting the importance of this pathway in longer-lived killifish. Our study demonstrates that other non-model nothobranchids can be used as comparative species to *N.*  *furzeri* in the study of aging, neurogenesis, and life history.

## Introduction

The African turquoise killifish *Nothobranchius furzeri* is a newly emerging model organism in the study of aging and age-dependent neurodegeneration due to its short lifespan (Isoe et al. 2012; Tozzini et al. 2012; Schmidt et al. 2013; de Bakker and Valenzano 2023). This species is found in east African countries such as Zimbabwe and Mozambique (Jubb 1971; Cellerino et al. 2016). The natural habitats of turquoise killifish are ephemeral pools formed by rainwater during the monsoon season, which typically last around 75 days (Tozzini et al. 2013). To survive such temporary and unpredictable environments, the turquoise killifish has evolved a two-phased life cycle (Vrtílek et al. 2018) that begins with hatching from embryos, followed by rapid growth that leads to sexual maturity in a few weeks. They reproduce and lay eggs in the mud that will enter diapause to survive through the dry season until the next monsoon arrives (Cellerino et al. 2016; Vrtílek et al. 2018).

The turquoise killifish maintains its annual life cycle even in captivity offering a convenient vertebrate model to study aging and age-related diseases (Terzibasi et al. 2007). Older turquoise killifish exhibits many hallmarks of aging including increased risk of cancer (Kim et al. 2016), spinal curvature (Cellerino et al. 2016), and telomere shortening (Hartmann et al. 2009). In the brain, characteristics of aging including learning impairments, gliosis, and reduced neurogenesis (Valenzano et al. 2006; Tozzini et al. 2012) have been observed. Teleost fishes such as medaka (*Oryzias latipes*) and zebrafish (*Danio rerio*) have long been used in the study of neurogenesis (Isoe et al. 2012; Schmidt et al. 2013), as fish possess much higher capacity to regenerate neurons compared to mammals (Zupanc 2006; Strobl-Mazzulla et al. 2010; Ganz and Brand 2016). The accelerated lifespan and neurodegenerative symptoms of *N. furzeri* make it an ideal model for investigating brain aging and rejuvenation (Vanhunsel et al. 2021; de Bakker and Valenzano 2023).

There are significant differences in longevity across strains of *N. furzeri* used in the laboratory, with a medium lifespan of 2–3 months in the GRZ strain and 6 months in the MZM-0403 strain (Anagnostopoulos et al. 2021). Additionally, there are even greater differences in lifespan within the family Nothobranchiidae including annual, non-annual, and semi-annual life histories. Annual species complete their life cycle within a year and produce eggs that go through diapause stages DI, DII, and DIII (Wourms 1972). Non-annual species typically live multiple (2–5) years in permanent water bodies and do not lay diapause eggs (Sahm et al. 2016). Semi-annualism/facultative annualism is thought to be the transitional life history between non-annualism and annualism (Hrbek and Larson 1999; Furness 2016). The developmental depressions in *F. gardneri* look like less intense versions of DII and DIII of the true diapause in *Austrafundulus limnaeus* and sometimes the embryos could skip the arrest and develop directly (Borisov et al. 2024). The variation of life histories and lifespans within the Nothobranchiidae makes them a unique group of fishes to study aging, evolution, life history strategies, and more (Reichwald et al. 2015).

Despite the growing research focus on *N. furzeri*, the other closely related nothobranchid species with divergent life histories remain understudied. For example, out of the 94 currently recognized *Nothobranchius* species (Nagy and Watters 2021), *Nothobranchius kuhntae* is the only other *Nothobranchius* species that has a partial genome assembly available. Additionally, *Aphyosemion australe* and *Callopanchax toddi* are the other nothobranchid species with genome assemblies on the National Center for Biotechnology Information (NCBI). Recently, the brain transcriptome of the longest living *Nothobranchius* species *N. guentheri* was sequenced and annotated (Guvatova et al. 2021). To our knowledge, no comparative brain transcriptomic study has yet been conducted on nothobranchid species across life histories.

In this study, we examined transcriptomic variation in the brains of seven nothobranchid species across three life histories using Quantseq 3′ mRNA sequencing (Moll et al. 2014). We conducted differential gene expression (DGE) analysis across life history comparisons (annuals vs. non-annuals, annuals vs. semi-annuals, and semi-annuals vs. non-annuals). Specifically, we aimed to investigate the expression of genes related to neurogenesis and aging in annual species compared to those with other life histories. We hypothesized that annual species would show downregulation in genes related to neurogenesis compared to non-annual and semi-annual species. Additionally, we conducted Gene Ontology (GO) enrichment and Kyoto Encyclopedia of Genes and Genomes (KEGG) pathway analyses to investigate what biological processes and pathways are enriched in annual species compared to other life histories. We hypothesized that longer-lived species, such as non-annuals and semi-annuals, would show enrichment in Notch signaling, a pathway involved in neurogenesis, compared to annual species. This hypothesis is based on findings that *N. furzeri* exhibits downregulation of the Notch signaling pathway in aging adults (Bagnoli and Terzibasi Tozzini 2021; Ayana et al. 2024).

This study builds on the work presented in Leow's master's thesis titled “Examining the Gene Expression Profiles of Killifishes With Different Life-History Using Liver and Brain Tissues (Cyprinodontiformes: Nothobranchiidae)” (Leow 2023).

## Methods and materials

The general methodology used in this study followed that of Leow and Piller (2024).

### Specimens and tissue collection

Seven species of nothobranchids (Table 1) across four genera and all life histories (annual, non-annual, and semi-annual) were obtained from aquarists and hobbyists. Three to six individuals were obtained from each species, and, when possible, equal numbers of males and females from each species were included in this study. Since the fish were acquired from aquarists and hobbyists, the exact age of the species was unknown. However, based on the life history traits, we estimated the annual *Nothobranchius* species were around 4–6 weeks old and non-annual *Fundulopanchax*, *Aphyosemion*, and *Epiplatys* species to be around 3–6 months old. To ensure all specimens were compared at the same developmental stage, which is sexually mature at young adult stage, juvenile/subadults were kept and raised in tanks until reaching adulthood before sacrificing the fish. All nothobranchids are sexually dimorphic and dichromatic, which made it easy to distinguish the sex and maturity of the specimens (Sedláček et al. 2014). When in captivity, fish were fed with frozen bloodworms and maintained at a constant water temperature and light/dark cycle (12 h each), following *N. furzeri* husbandry in research protocols (Astre et al. 2022) with some modifications. Adult fish were euthanized using tricaine methanesulfonate (MS-222) following the requirement of Institutional Animal Care and Use Committee standards for fish (American Association for Laboratory Animal Sciences) and SLU-IUCAC #2 at Southeastern Louisiana University. Liver tissues were preserved in RNAlater (Qiagen) immediately upon harvesting and stored at −80°C until RNA isolation. The remaining carcasses were preserved in RNAlater and stored at −80°C.

**Table 1:** Nothobranchid species used in this study^a^

**Species**	**Symbol**	**Life history**	**Males**	**Females**	**Total**
*Nothobranchius rubripinnis*	NRb	Annual	4	2	6
*Nothobranchius rachovii*	NRv	Annual	2	3	5
*Nothobranchius eggersi*	NE	Annual	3	3	6
*Fundulopanchax gardneri*	FG	Semi-annual	3	3	6
*Aphyosemion splendopleure*	Asp	Non-annual	3	3	6
*Aphyosemion bivittatum*	AB	Non-annual	3	3	6
*Epiplatys guineensis*	EG	Non-annual	3	0	3
			19	17	38

^a^There are a total of 38 individuals from four genera and seven species.

### RNA isolation and sequencing

Total RNA was isolated from the liver tissue with RNeasy Plus Universal Mini Kit (Qiagen) following the standard protocol (revised October 2020). The quality of RNA was checked by visualizing the RNA product on a 2% agarose gel. RNA concentrations were measured using Qubit 4 Fluorometer (Thermo Fisher Scientific). Five hundred nanograms of RNA from each sample was prepared and sent to Iowa State University DNA Facility for library prep and sequencing. QuantSeq (Moll et al. 2014) was chosen over traditional RNA-Seq due to our large sample size and cheaper library preparation cost for QuantSeq. Samples were sequenced using an SP flow cell for 100-bp single-end reads on NovaSeq 6000.

### Reads processing and mapping

Raw sequence reads were downloaded and checked with FastQC (Andrews 2010) for quality. Reads were trimmed to remove the first 12 bp due to low quality (-u 12), poly-A tail (-n 3 -a “A{20}”), low quality reads (Phred score less than 25), and reads less than 20 bp long (-m 20) using cutadapt v.4.1 (Martin 2011). Trimmed reads were once again checked with FastQC for quality. The turquoise killifish genome (GCA_001465895.2) and the GFF annotation file were downloaded from the NCBI website. All reads were aligned to the turquoise killifish genome (Valenzano et al. 2015) using STAR 2.7.10a (Dobin et al. 2013). A genome index was created from the annotated turquoise killifish genome for downstream mapping. A two-pass mapping approach was used as recommended in the STAR manual for a study with multiple samples (Dobin et al. 2013). The first mapping pass was run for all samples with the default parameters and the junctions were collected. The second mapping pass was run for all samples, listing SJ.out.tab files from all samples generated in the first mapping. The number of reads per gene was counted using –quantMode simultaneously during the second mapping with the following parameters as suggested by QuantSeq mapping protocol –outFilterMultimapNmax 20 –alignSJoverhangMin 8 –alignSJDBoverhangMin 1 –outFilterMismatchNmax 999–outFilterMismatchNoverLmax 0.6–alignIntronMin 20 –alignIntronMax 1000000–alignMatesGapMax 1000000. Read counts of all samples from the third column in the ReadsPerGene.out.tab files were concatenated into an excel file before importing into R (R Core Team 2022) and RStudio v.4.2.2. (RStudio Team 2020) for analysis and visualization. Raw sequence reads were deposited to NCBI Sequence Read Archive (SRA) under project PRJNA1178144.

### Differential gene expression analysis

Low count loci (reads sum <10) were filtered out to reduce the memory size of the data object and increase the speed of downstream analyses. Size factors were considered for different library sizes. DGE analyses were conducted using the R package DESeq2 v.1.36.0 (Love et al. 2014). DGE analyses were conducted in pairwise comparison between life histories: annual versus non-annual (A vs. N), annual versus semi-annual (A vs. SA), and semi-annual versus non-annual (SA vs. N). The false discovery rate cutoff (alpha) was set to 0.05 and the log2FoldChange was set to 1 (a two-fold change) to detect differentially expressed genes (DEGs). We took a three-pronged approach to examine DGE across life histories. First, we examined the 500 most variable genes to give a broad perspective of DGE across life histories using principal component analysis (PCA). Volcano plots of the DEGs (pairwise comparisons between life histories) were made using function *produce volcano* from an R package rnaseq v.1.0.8. (Beauparlant 2022).

### GO enrichment and KEGG pathway analyses

All the significant DEGs were separated into an upregulated gene list and downregulated gene list from each pairwise comparison. Hong et al. (2013) noted that analyzing up- and downregulated genes separately is more powerful to detect meaningful GO terms than analyzing all DEGs simultaneously. These gene lists were then uploaded separately to the DAVID 2021 webserver (Huang et al. 2009) for GO term enrichment analysis and KEGG pathway analysis. When necessary, genes submitted to the DAVID webserver were programmatically converted from official gene symbols to entrez gene IDs by DAVID. Gene IDs that were not convertible in the DAVID database were filtered out and the remaining genes were submitted to DAVID with *N. furzeri* as background. An EASE score of 0.05, a modified Fisher Exact *P* value for gene enrichment analysis, and a gene count of 2 (default by DAVID) were used to determine significantly enriched GO terms and KEGG pathways. The GO terms from biological process direct and the associated *P* values were uploaded to REVIGO (Supek et al. 2011) to summarize them by removing redundant GO terms. X and Y axes are sematic similarity, which circles cluster together are more closely related. Circle color and size indicate log10 *P* value of each GO term (Fig. 5A–D). The threshold of the EASE score, a modified Fisher exact *P* value for gene enrichment analysis was set to 0.05. All GO terms and the associated *P* values were run through REVIGO with the same settings: removed obsolete GO terms, searched against the whole UniProt database (turquoise killifish was not an option), and used SimRel as sematic similarity measure.

## Results

### Sequence reads

**Fig. 1:**
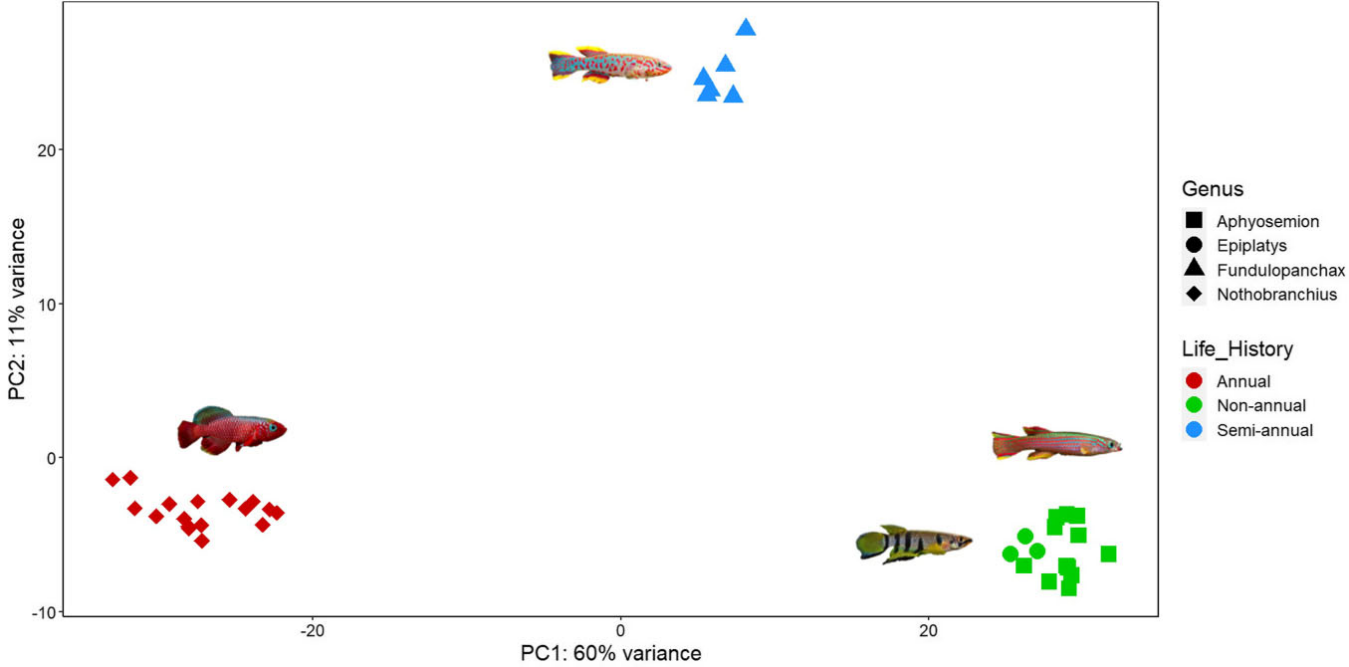
Principal component analysis of the 38 sequenced nothobranchids (brain tissue) based on the top 500 most variable genes. Shapes represent the four genera and colors represent different life histories.

We obtained 516.97 million reads from the 40 individuals, averaging 13.25 million reads per sample. Samples EG4 and NRvm1 were discarded due to poor quality reads. After trimming with cutadapt (Martin 2011), the remaining 38 individuals were mapped to the closest available annotated reference genome *N. furzeri* (GCA_001465895.2) using STAR (Dobin et al. 2013). The percentage of reads mapped can be found in Supplementary Material (Supplementary Table 7). PCA was conducted on the 38 individuals to examine overall gene expression variation (500 most variable genes) in multivariate space (Fig. 1). The analysis recovered three non-overlapping clusters, with the first two principal component (PC) axes accounting for 71% of the total variance (PC1 = 60% and PC2 = 11%). The first distinct cluster consists of all annual species including *N. rubripinnis*, *N. rachovii*, and *N. eggersi*. The second cluster contains the only semi-annual species included in the study, *F. gardneri*. The third cluster includes non-annual species including *E. guineensis*, *A. splendopleure*, and *A. bivittatum*. The brain transcriptomic profiles of these nothobranchid species clustered by life histories. We compared the DEGs between *Fundulopanchax* and *Aphyosemion* and found that *F. gardneri* exhibited 847 uniquely upregulated and 735 downregulated genes in the brain compared to the liver (Fig. 7). GO and KEGG pathway analyses showed that *F. gardneri* is most enriched in mitosis, cell division, and cell cycle (Fig. 7). Raw results can be found in Supplementary Table 5.

### Differential gene expression analysis

**Fig. 2:**
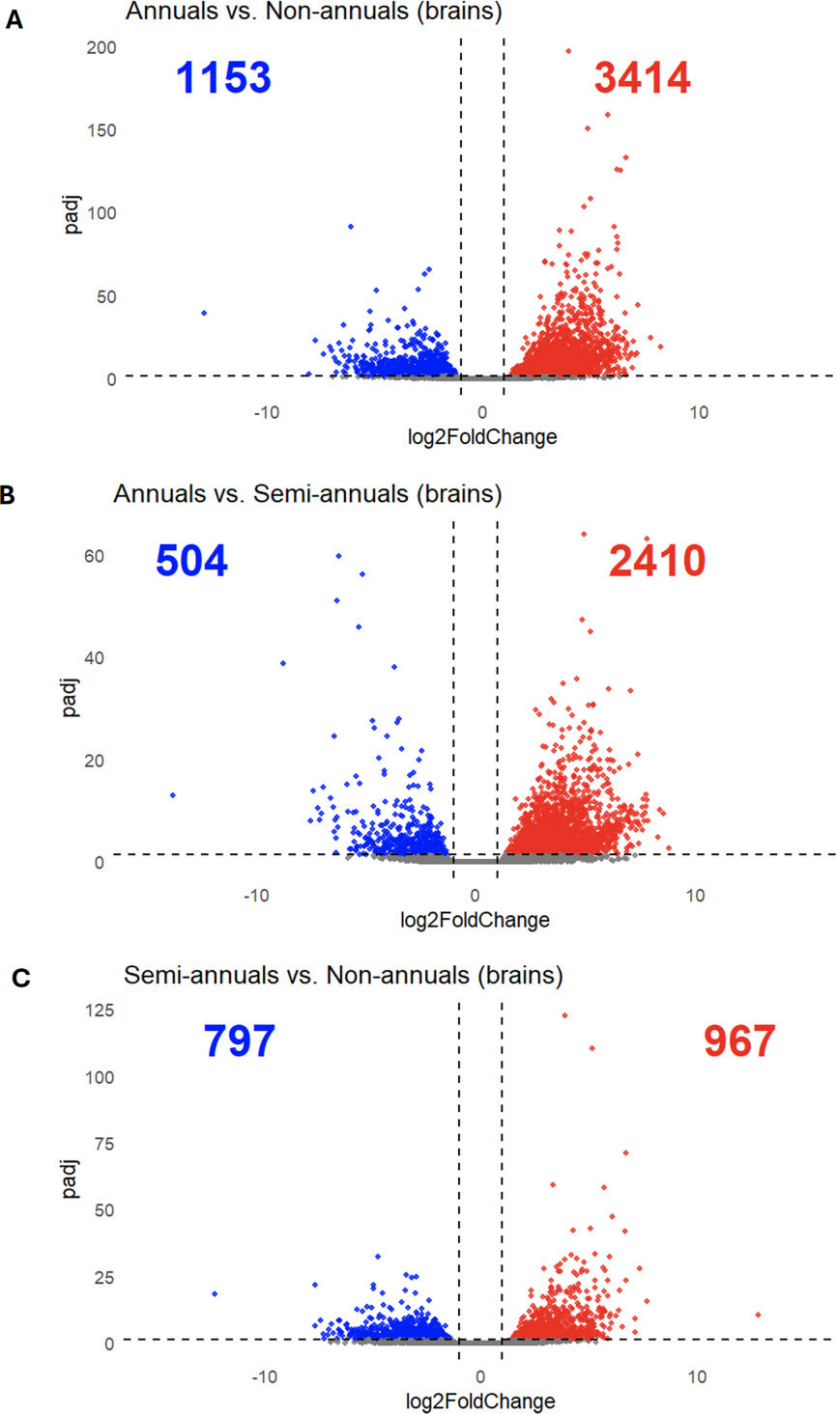
Volcano plots of the differentially expressed genes among life histories (brain): (**A**) annuals versus non-annuals, (**B**) annuals versus semi-annuals, and (**C**) semi-annuals versus non-annuals. Red represents significantly upregulated genes. Blue represents significantly downregulated genes.

The gene expression patterns were compared across life histories (annuals vs. non-annuals, annuals vs. semi-annuals, and semi-annuals vs. non-annuals) with a two-fold change in expression level and a false discovery rate below 0.05. There are 3414 upregulated genes and 1153 downregulated genes in annuals compared to non-annuals (Fig. 2). There are 2410 upregulated genes and 504 downregulated genes in annuals compared to semi-annuals. Finally, there are 967 upregulated genes and 797 downregulated genes in semi-annuals compared to non-annuals.

**Fig. 3:**
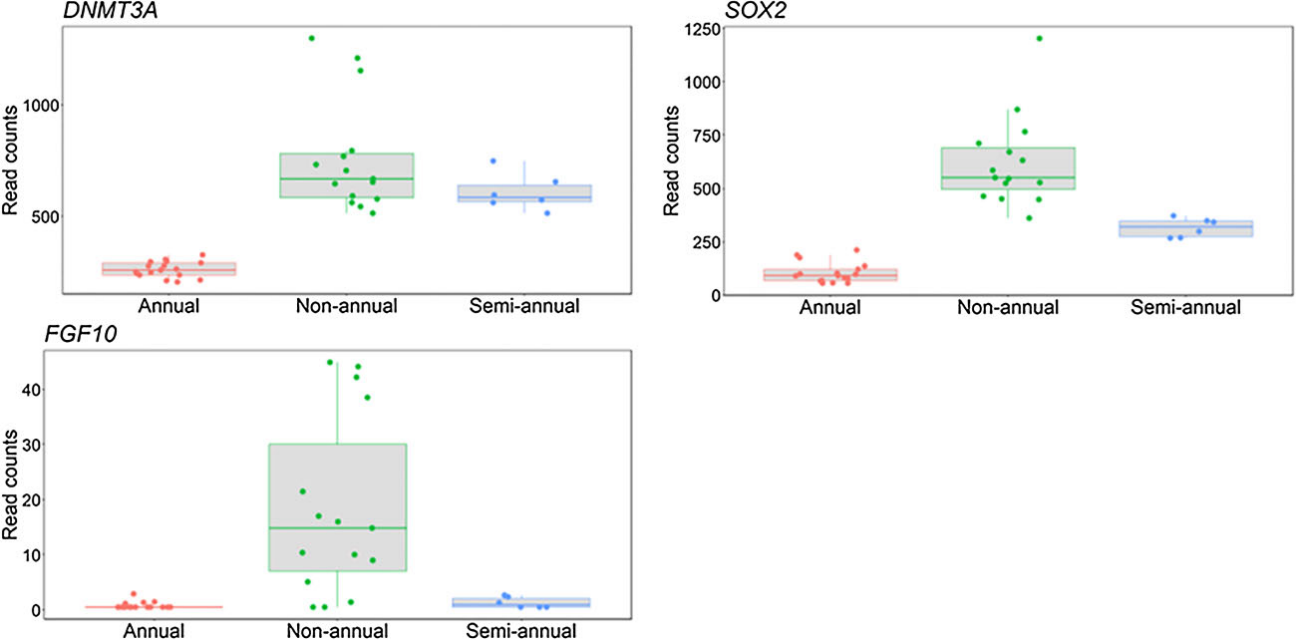
Plot count of genes *DNMT3A*, *SOX2*, and *FGF10* between life history comparisons.

**Fig. 4:**
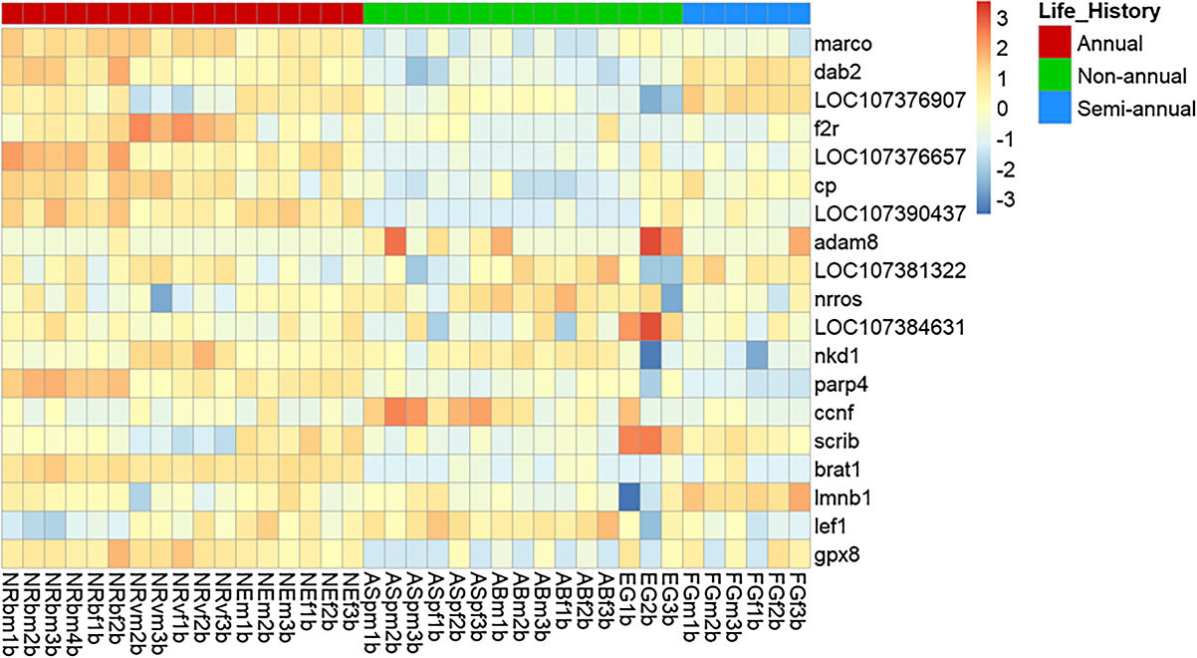
Heatmap of genes showed conserved aging expression signatures across tissues and species in our study, described in Barth et al. (2019). Some of the described genes were not found in our reference genome. Gene loci corresponding to genes are as follows: LOC107376907: *cyp26b1*, LOC107376657: *lgals1*, LOC107390437: cd40, LOC107381322: *cyba*, and LOC107384631: *cybb*.

From the DEGs among life history comparisons, we specifically examined 26 genes that showed conserved aging expression signatures across tissues (brain, liver, and skin) and species (*Homo sapiens*, *Mus musculus*, *Danio rerio*, and *N. furzeri*) described in Barth et al. (2019). Out of the 26 genes, we identified 19 in our transcriptomic data. Seven genes, *SH2D1B1*, *PTAF2*, *PDGFRB*, *CDKN2B*, *RASSF4*, *CSCA8*, and *BIRC5* were not identified in our data. Our results showed that all 19 genes were upregulated in annual species compared to other life histories (Fig. 4). Among the 1153 downregulated genes in annual species, we found that SRY-box transcription factor 2 (*SOX2*), DNA methyltransferase 3 alpha (*DNMT3A*), and fibroblast growth factor 10 (*FGF10*) were downregulated in annuals compared to non-annuals and semi-annuals (Fig. 3).

### GO enrichment analysis

To understand what biological processes are enriched in these DEGs, we conducted GO enrichment analysis using the DAVID Bioinformatics Resources webserver (Huang et al. 2009). The significant DEGs in each life history comparison were separated into upregulated and downregulated gene lists before conducting GO enrichment analysis. Previous research suggested that this was a better way than analyzing all DEGs together for detecting meaningful enrichment (Hong et al. 2013).

**Fig. 5:**
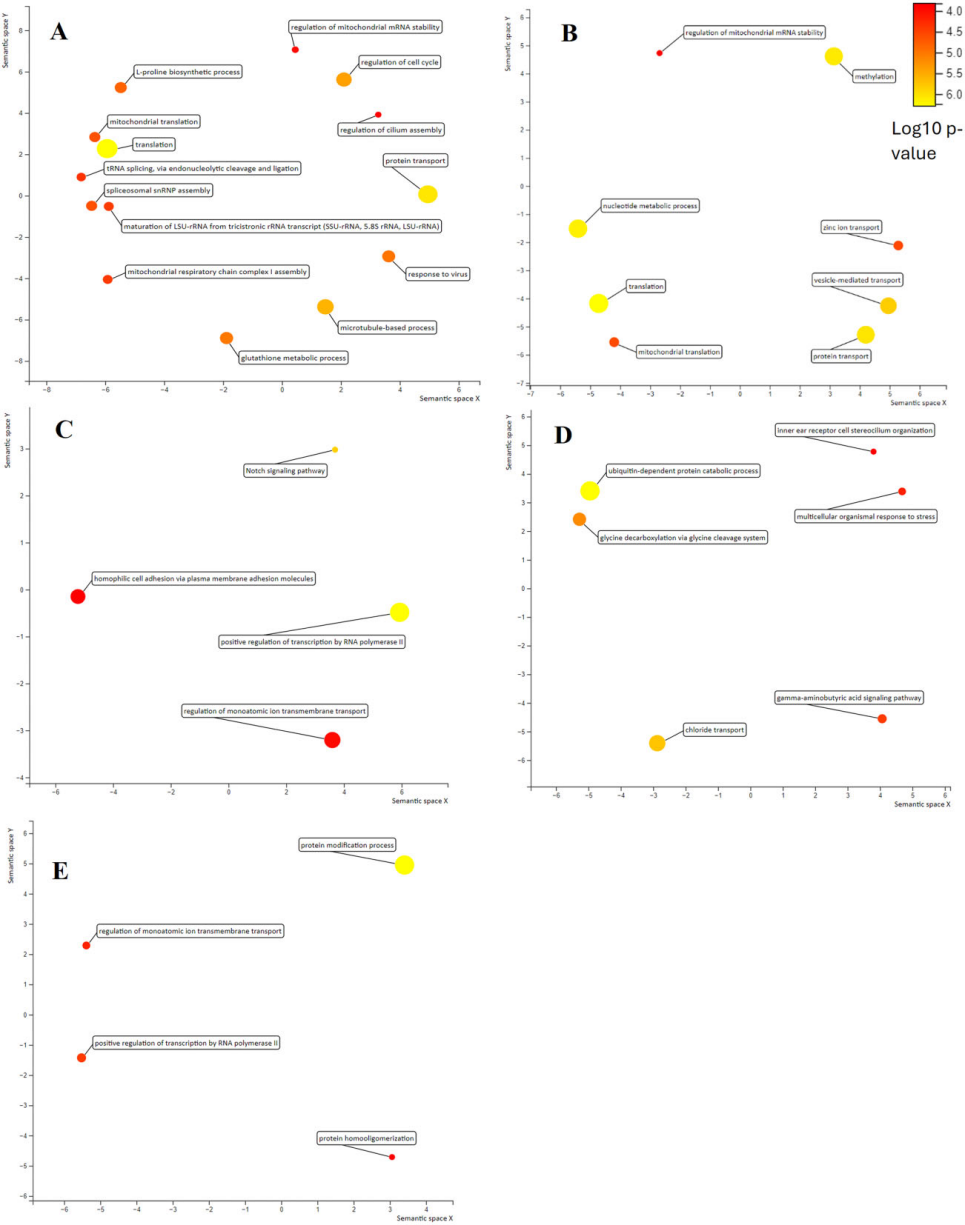
Enriched GO terms visualized in multidimensional scaling plots: (**A**) annuals versus non-annuals, (**B**) annuals versus semi-annual, (**C**) non-annuals versus annuals, (**D**) semi-annuals versus annuals, and (**E**) non-annuals versus semi-annuals. X and Y axes are sematic similarities.

Annuals are enriched in 14 GO biological process terms compared to non-annuals including translation, protein transport, spliceosomal snRNP assembly, mitochondrial translation, regulation of cilium assembly, response to virus, glutathione metabolic process, mitochondrial respiratory chain complex I assembly, maturation of LSU-rRNA, l-proline biosynthetic process, regulation of mitochondrial mRNA stability, regulation of cell cycle, microtubule-based process, and tRNA splicing (Fig. 5). Annuals are enriched in eight GO terms compared to semi-annuals including protein transport, vesicle-mediated transport, regulation of mitochondrial mRNA stability, mitochondrial translation, translation, zinc II ion transport, methylation, and nucleotide metabolic process (Fig. 5A). Non-annuals are enriched in five GO terms compared to annuals including the Notch signaling pathway, positive regulation of transcription from RNA polymerase II promoter, regulation of ion transmembrane transport, and homophilic cell adhesion via plasma membrane adhesion molecules (Fig. 5B). The DAVID webserver identified several genes involved in the Notch signaling pathway that were differentially expressed between annuals and non-annuals (Fig. 5C). Semi-annuals are enriched in six GO terms compared to annuals including gamma-aminobutyric acid signaling pathway, chloride transport, glycine decarboxylation via glycine cleavage system, ubiquitin-dependent protein catabolic process, multicellular organismal response to stress, and inner ear receptor stereocilium organization (Fig. 5C). Non-annuals are enriched in four GO terms compared to semi-annuals including cellular protein modification process, protein homo-oligomerization, regulation of ion transmembrane transport, and positive regulation of transcription from RNA polymerase II promoter (Fig. 5D). No significant GO terms were enriched in semi-annuals compared to non-annuals.

### KEGG pathway analysis

KEGG pathway analysis was performed on the DEGs between annuals and other life histories using the DAVID webserver (Huang et al. 2009). Sixteen enriched KEGG pathways were found in annuals compared to non-annuals (Table 2). The top three KEGG pathways are ribosome, metabolic pathways, and oxidative phosphorylation. Thirteen KEGG pathways were enriched in annuals compared to semi-annuals. Among these 13 pathways, the top three are metabolic pathways, ribosome biogenesis in eukaryotes, and aminoacyl-tRNA biosynthesis (Table 3).

**Table 2:** KEGG: annuals versus non-annuals^a^

**Term**	**Count**	**%**	***P*** **value**
nfu03010:Ribosome	54	1.681	5.68E-15
nfu01100:Metabolic pathways	309	9.620	7.87E-09
nfu00190:Oxidative phosphorylation	37	1.152	6.59E-05
nfu01232:Nucleotide metabolism	29	0.903	2.46E-04
nfu00240:Pyrimidine metabolism	20	0.623	8.72E-04
nfu04146:Peroxisome	25	0.778	0.00152
nfu05168:Herpes simplex virus 1 infection	82	2.553	0.00216
nfu01200:Carbon metabolism	30	0.934	0.00507
nfu04621:NOD-like receptor signaling pathway	33	1.027	0.01528
nfu00230:Purine metabolism	33	1.027	0.02138
nfu04623:Cytosolic DNA-sensing pathway	12	0.374	0.02509
nfu03020:RNA polymerase	10	0.311	0.02765
nfu03030:DNA replication	11	0.342	0.03146
nfu00730:Thiamine metabolism	6	0.187	0.04223
nfu01240:Biosynthesis of cofactors	32	0.996	0.04625

^a^Enriched pathways obtained from submitting the DEGs to the DAVID webserver. Threshold of minimum gene counts 2 (belonging to an annotation term) and EASE score threshold 0.05 were used to determine significant KEGG pathways.

**Table 3:** KEGG: annuals versus semi-annuals^a^

**Term**	**Count**	**%**	***P*** **value**
nfu01100:Metabolic pathways	204	9.003	4.41E-06
nfu03008:Ribosome biogenesis in eukaryotes	18	0.794	4.87E-04
nfu00970:Aminoacyl-tRNA biosynthesis	11	0.485	0.00458
nfu01232:Nucleotide metabolism	19	0.838	0.00506
nfu03020:RNA polymerase	9	0.397	0.00721
nfu04146:Peroxisome	17	0.750	0.00946
nfu04623:Cytosolic DNA-sensing pathway	10	0.441	0.01219
nfu04145:Phagosome	24	1.059	0.01697
nfu04142:Lysosome	25	1.103	0.01705
nfu05168:Herpes simplex virus 1 infection	53	2.339	0.02225
nfu01200:Carbon metabolism	20	0.883	0.02491
nfu00860:Porphyrin metabolism	8	0.353	0.03252

^a^Enriched pathways obtained from submitting the DEGs to the DAVID webserver. Threshold of minimum gene counts 2 (belonging to an annotation term) and EASE score threshold 0.05 were used to determine significant KEGG pathways.

## Discussion

### Global gene expression analysis

The gene expression profiles of nothobranchid brains clustered by life history in the PCA (Fig. 1). This contrasts with our previous findings, based on liver tissue from the same individual specimens, which showed that the gene expression profiles of nothobranchid species clustered according to their phylogenetic relationships (fig. 1 in Leow and Piller 2024). In this study, each of the first two clusters included the annual (*Nothobranchius*) and semi-annual (*Fundulopanchax*) genera, while the third cluster comprised two non-annual genera, *Epiplatys* and *Aphyosemion*. Although *Fundulopanchax* and *Aphyosemion* are more closely related to each other than *Epiplatys*, in our results, *Aphyosemion* is grouped with *Epiplatys*. A detailed phylogeny of Nothobranchiidae is described in Thompson et al. (2024). Notably, the *Fundulopanchax* cluster is positioned between the annual and non-annual clusters, suggesting an intermediate life history between the two (Fig. 1). To investigate what genes might be contributing to the separation of expression profiles between the semi-annual *F. gardneri* and non-annual *Aphyosemion* species, we compared the DEGs between brain and liver tissues (Leow and Piller 2024). We found that the *F. gardneri* brain exhibited 847 uniquely upregulated and 735 downregulated genes compared to *Aphyosemion* species (Fig. 7). GO and KEGG pathway analyses on the upregulated genes showed enrichment in mitosis, cell division, cell cycle, and others (Fig. 7). This suggests that a faster cell cycle may be driving the gene expression in semi-annual brain compared to the closely related *Aphyosemion* genus*.* To gain a more comprehensive understanding of the transcriptomics associated with a semi-annual life history, future studies should include additional *Fundulopanchax* species to provide a more robust representation of semi-annual species.

A study done by Sowersby et al. (2021) showed that annual killifish species in Aplocheiloidei (includes Nothobranchiidae and Rivulidae) have larger brain size compared to non-annual species. They suggested that the ephemeral habitats in which the annual species occupy can impose higher cognitive demands and therefore favor larger brain sizes, a pattern that also has been observed in birds and primates (van Woerden  et al. 2012; Sayol et al. 2016). In our study, these differences in cognitive capacity across life histories may drive gene expression patterns in nothobranchid brains. Additionally, in our study, annuals exhibited the most upregulated and downregulated genes compared to non-annual and semi-annual species (Fig. 2), which is consistent with our previous findings in the liver tissues (Leow and Piller 2024).

### Genes associated with neurogenesis downregulated in annuals

We found that genes related to neurogenesis *SOX2*, *DNMT3A*, and *FGF10* showed downregulation in annuals compared to non-annuals (Fig. 3). *DNMT3A* is a chromatin remodeling gene involved in epigenetic modification in development (Bird 2002) and neurogenesis, and is downregulated with age in *N. furzeri* (Baumgart et al. 2014). *SOX2*, a gene conserved in evolution, is critical for its functional relevance in stem cells (Mercurio et al. 2019). *FGF10* promotes proliferation of neural progenitors and is upregulated after neuronal damage (Yoshimura et al. 2001; Guillemot and Zimmer 2011). The accelerated aging phenotypes observed in the annual life history might be driving the downregulation of neurogenesis in the adult brains, leading to downregulation of these genes. However, the early adult stage of the annual killifish might not be exhibiting signs of neurodegeneration yet.

### Conserved aging-related genes

We examined 26 genes that displayed similar expression patterns during aging across different tissues (brain, liver, and skin) in multiple species (*H. sapiens*, *M. musculus*, *D. rerio*, and *N. furzeri*), as identified by Barth et al. (2019). From these 26 genes, we identified 19 in our transcriptomic data, all of which showed upregulation in annuals compared to non-annuals and semi-annuals. Barth et al. (2019) also noted that longer-lived species exhibited more controlled gene expression variance compared to short-lived species like *N. furzeri*. The looser control in gene expression of the annual species might explain the upregulation in these 19 age-related genes. Notably, our study only compares gene expression patterns across life history at the early adult stage rather than aging stages, which may explain the different results we observed.

### GO enrichment analysis

We conducted GO enrichment analysis to investigate the GO terms and pathways enriched in each life history comparison. The top four enriched biological process GO terms in annuals compared to non-annuals are translation, protein transport, spliceosomal snRNP assembly, and mitochondrial translation. These four GO terms are also enriched in the liver tissues of annual species from our previous findings in the liver tissues (Leow and Piller 2024). Since the liver and brain have very different functions, these enriched functional GO terms are likely the result of the accelerated annual life history of *Nothobranchius* spp. Indeed, Reichwald et al. (2015) discovered that translational and ribosomal processes are enriched in *N. furzeri* during both the diapause and brain aging phases. Similar enriched GO terms are also found in elderly *N. furzeri* and human brains, including pathways related to translational elongation, protein targeting to ER, and ribosomes (Baumgart et al. 2014).

One of the 14 enriched GO biological process terms in annual species is mitochondrial respiratory chain complex I assembly. Mitochondrial complex I is the main entry point for electrons into the electron transport chain, which is required for ATP production via oxidative phosphorylation (Sharma et al. 2009; Formosa et al. 2018). In an RNA-Seq study, Baumgart et al. (2016) identified mitochondrial respiratory chain complex I as a hub in a group of genes, whose expression is negatively correlated with lifespan. Our results indicate that in the brains of the annual species, mitochondrial respiratory chain complex I assembly is enriched compared to non-annual species, supporting the idea that the expression of this pathway is negatively correlated with longevity.

### Notch signaling pathway in the non-annual killifish

**Fig. 6:**
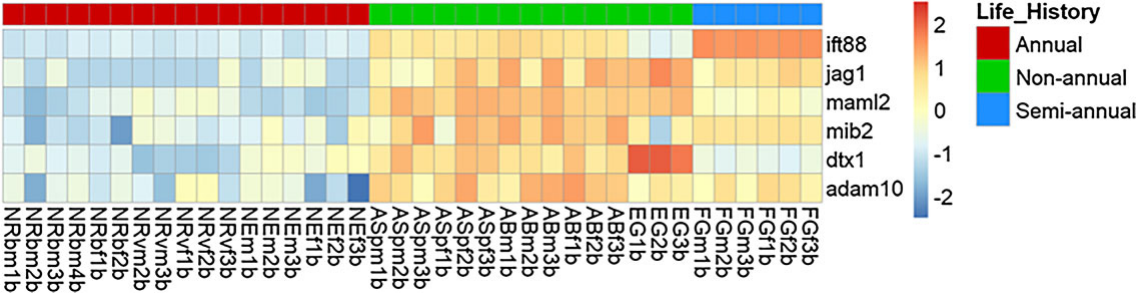
Genes involved in the Notch signaling pathway identified by the DAVID webserver that are downregulated in annuals compared to other life histories.

The Notch signaling pathway is a conserved pathway that is important in the development of many tissues (Balistreri et al. 2016). In the brain tissue, the Notch signaling pathway is associated with adult neurogenesis in vertebrates, including zebrafish and turquoise killifish (Than-Trong et al. 2018; Sueda and Kageyama 2020; Bagnoli and Terzibasi Tozzini 2021) and acts as a master regulator of adult neurogenesis in mammalian brains (Lampada and Taylor 2023). Non-annual species are enriched in the Notch signaling pathway compared to annual species (Fig. 5C). The DAVID webserver identified that several genes (*IFT88*, *JAG1*, *MAML2*, *MIB2*, *DTX1*, and *ADAM10*) involved in the Notch signaling pathway were downregulated in annuals compared to non-annuals (Fig. 6). This is consistent with recent studies (Bagnoli and Terzibasi Tozzini 2021; Ayana et al. 2024), where they found that *N. furzeri* shows an age-dependent downregulation of the Notch signaling pathway in brain tissue. Although we did not find GO neurogenesis term enriched in non-annuals, the upregulation of the Notch signaling pathway suggests non-annuals might have higher neurogenesis capacity at the same developmental stage compared to annuals. Similar to our previous liver transcriptomic study, we found that the Notch signaling pathway was enriched in the non-annual species (Leow and Piller 2024). Since the liver and brain have distinct functions, it is likely that the Notch signaling pathway plays an important role in the longer-lived non-annual species. Future studies should investigate the expression of the Notch signaling pathway throughout the life cycle of non-annual or semi-annual species—such a study may require raising fish from embryos.

**Fig. 7:**
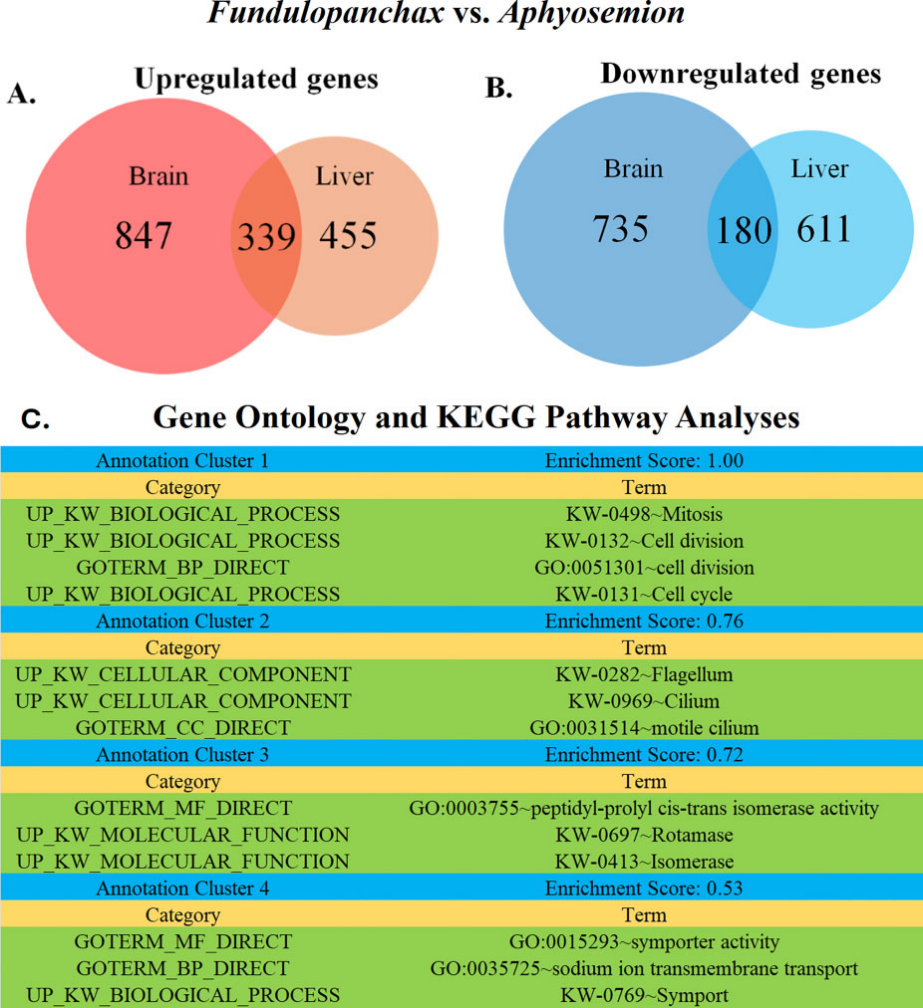
Venn diagrams of unique differentially expressed genes in semi-annual *Fundulopanchax* versus non-annual *Aphyosemion*. (**A**) Upregulated genes in the brain versus liver and (**B**) downregulated genes in the brain versus liver. Full lists of genes are shown in Supplementary Material (Supplementary Table 4). (**C**) GO and KEGG pathway analyses of the uniquely upregulated genes in *Fundulopanchax* versus *Aphyosemion* brain.

### KEGG pathway analysis

Most of the enriched KEGG pathways identified in annual species are related to metabolism (Tables 2 and 3). Notably, two of the most enriched pathways (first and third) in annuals versus non-annuals are ribosome and oxidative phosphorylation (Table 2). Similarly, the second most enriched pathway in annuals versus semi-annuals is ribosome biogenesis in eukaryotes (Table 3). Baumgart et al (2016) found that longer-lived *N. furzeri* showed lower expressions in oxidative phosphorylation and ribosome pathways compared to short-lived individuals. Here, we show that longer-lived non-annuals also display lower expression levels of these two pathways compared to annuals.

## Conclusion

In this study, we examined the brain transcriptomic signatures of young adult nothobranchid killifish across life histories. We identified many DEGs, and our results show that life histories have a significant impact on the gene regulations in the killifish brain.

Using non-model organisms in research offers both advantages and challenges. One caveat is the lack of reference genomes. Currently *N.*  *furzeri* has the most well-annotated reference genome among nothobranchid killifish. While we acknowledge the potential mapping bias introduced by aligning sequencing reads to a single reference genome, this was the best option for our study. As sequencing costs decrease and genome assembly becomes more affordable, we anticipate the availability of more reference genomes for killifish in the near future.

Though using pet trade-sourced organisms is convenient, obtaining the exact age of the fish can be difficult or even impossible. Raising fish from embryos would provide the most comprehensive understanding of life history, but the time and resources required are significant trade-offs.

Model organisms are invaluable in advancing our understanding of biology, and short-lived species such as *N. furzeri* and its relatives are emerging as powerful and convenient research systems. Mazzetto et al. (2022) found that the brains of wild and captive *N. furzeri* show the same direction of regulation in the DEGs. Therefore, we believe our results are comparable to those from wild killifish and contribute valuable insights into the effects of life history on brain transcriptomics. Combined with existing research on *N. furzeri*, our study lays the groundwork for using other non-model nothobranchid species in the study of life history, aging, and neurobiology.

## Supplementary Material

obaf016_Supplemental_File

## Data Availability

Raw sequence reads were deposited to NCBI Sequence Read Archive (SRA) under project PRJNA1178144. Supplementary tables and data can be found in Microsoft Excel Supplementary_Material.
